# Facioscapulohumeral dystrophy in children: design of a prospective, observational study on natural history, predictors and clinical impact (iFocus FSHD)

**DOI:** 10.1186/s12883-016-0664-6

**Published:** 2016-08-17

**Authors:** Rianne J. M. Goselink, Tim H. A. Schreuder, Karlien Mul, Nicol C. Voermans, Maaike Pelsma, Imelda J. M. de Groot, Nens van Alfen, Bas Franck, Thomas Theelen, Richard J. Lemmers, Jean K. Mah, Silvère M. van der Maarel, Baziel G. van Engelen, Corrie E. Erasmus

**Affiliations:** 1 Department of Neurology, Donders Center for Neuroscience, Radboud University Medical Center, Nijmegen, The Netherlands; 2 Department of Rehabilitation, Donders Center for Neuroscience, Radboud University Medical Center, Nijmegen, The Netherlands; 3 Department of Clinical audiology, Radboud University Medical Center, Nijmegen, The Netherlands; 4 Department of Op Ophthalmology, Radboud University Medical Center, Nijmegen, The Netherlands; 5 Department of Human Genetics, Leiden University Medical Center, Leiden, The Netherlands; 6 Department of Paediatric Neurology, Alberta Children’s Hospital, Calgary, Canada

**Keywords:** Facioscapulohumeral dystrophy, Neuromuscular diseases, Muscular dystrophies, Paediatrics, Observational study

## Abstract

**Background:**

Facioscapulohumeral muscular dystrophy (FSHD; OMIM 158900 & 158901) is a progressive skeletal muscle dystrophy, characterized by an autosomal dominant inheritance pattern. One of the major unsolved questions in FSHD is the marked clinical heterogeneity, ranging from asymptomatic individuals to severely affected patients with an early onset. An estimated 10 % of FSHD patients have an early onset (onset before 10 years of age) and are traditionally classified as infantile FSHD. This subgroup is regarded as severely affected and extra-muscular symptoms, such as hearing loss and retinopathy, are frequently described. However, information on the prevalence, natural history and clinical management of early onset FSHD is currently lacking, thereby hampering adequate patient counselling and management. Therefore, a population-based prospective cohort study on FSHD in children is highly needed.

**Methods/design:**

This explorative study aims to recruit all children (aged 0–17 years) with a genetically confirmed diagnosis of FSHD in The Netherlands. The children will be assessed at baseline and at 2-year follow-up. The general aim of the study is the description of the clinical features and genetic characteristics of this paediatric cohort. The primary outcome is the motor function as measured by the Motor Function Measure. Secondary outcomes include quantitative and qualitative description of the clinical phenotype, muscle imaging, genotyping and prevalence estimations. The ultimate objective will be a thorough description of the natural history, predictors of disease severity and quality of life in children with FSHD.

**Discussion:**

The results of this population-based study are vital for adequate patient management and clinical trial-readiness. Furthermore, this study is expected to provide additional insight in the epigenetic and environmental disease modifying factors. In addition to improve counselling, this could contribute to unravelling the aetiology of FSHD.

**Trial registration:**

clinicaltrials.gov NCT02625662.

## Background

Facioscapulohumeral dystrophy (FSHD) is a muscular dystrophy characterized by progressive weakness and atrophy of the facial (facio-), shoulder-upper arm (scapulohumeral-), axial- and leg muscles [1–3]. FSHD is one of the most prevalent muscular dystrophies with an estimated prevalence of 12:100.000 [4].

One of the hallmarks of FSHD is its clinical heterogeneity; the spectrum varies from severely affected, wheelchair bound children to asymptomatic carriers in late adulthood, even within families with the same repeat contraction [1, 5, 6]. Typically FSHD has an onset in adolescence and life expectancy is not impaired [7]. However, a subgroup of patients has a childhood onset and is associated with more severe disease progression. FSHD is increasingly recognized as an epigenetic disease, which could be an explanation for this clinical heterogeneity.

### Classification

Traditionally, children with very early onset have been classified as a distinct disease identity named *infantile FSHD* [8], based on the following criteria [9]:

- signs or symptoms of facial weakness before the age of 5 and
- signs or symptoms of scapular weakness before the age of 10

The concept of two FSHD types has gradually developed to an FSHD spectrum, with infantile onset being on the severe end and asymptomatic carriers on the other end. Accordingly, recent articles mostly classify the disease severity according to the repeat length of the genetic defect in FSHD1 [10–12]. However, the correlation between disease severity and repeat length is inconsistent and is influenced by other genetic and environmental modifiers [13–15]. A generally accepted definition of severely affected FSHD or early onset “classical” patients is currently lacking.

### Prevalence

Few studies have investigated the prevalence of childhood onset FSHD and these studies have used different selection criteria. Estimations of early onset FSHD vary between 3–21 % of the total FSHD population [1, 16] and 58 % of the pediatric FSHD population (onset at any age < 18 years [17]). Accurate prevalence estimations of childhood onset FSHD are useful for interpretation of clinical studies, planning and increasing clinical trial-readiness.

### Extramuscular symptoms

Various extramuscular symptoms such as epilepsy, hearing difficulties, retinal abnormalities (Coats’ syndrome), mental retardation and cardiac arrhythmias are associated with FSHD and they are most frequently described in the early onset subgroup [9–12, 17–19]. Little is known regarding the prevalence and aetiology of these extramuscular symptoms in children with FSHD. The current body of knowledge may be skewed by publication bias, selection bias and no/incorrect molecular diagnoses.

### Genetics

FSHD is increasingly recognized as an epigenetic disease. The most frequent cause of FSHD is contraction of the polymorphic D4Z4 macrosatellite repeat array in the subtelomere of chromosome 4 at 4q35 resulting in FSHD type 1 (FSHD1) [20]. This mutation explains >95 % of the adult cases and all known infantile cases. Healthy individuals have 8 or more D4Z4 repeats on each 4q35 copy, whereas patients with FSHD1 have 1–10 repeats on one copy of the 4q35 chromosome region and a disease-permissive allele 4A on the chromosome 4q subtelomere. In contrast to 4A alleles, 4B alleles, which lack a polyadenlylation signal for the *DUX4* gene which is embedded in each repeat of the D4Z4 array, are non-permissive to the disease [21]. There is a copy of the D4Z4 repeat array on chromosome 10, but in absence of a DUX4 polyadenylation signal, contractions of this array are also not pathogenic [22].

This causative genotype explains the variability in disease onset and progression only to some extent. The inverse correlation between residual repeat length and disease severity (short repeat lengths are associated with an earlier onset, wheelchair dependency and extramuscular involvement [23, 24]) is imperfect; for example, it does not explain the intra-familial differences in phenotype. Aberrant epigenetic regulation of the D4Z4 chromatin structure is thought to play an important role in this clinical heterogeneity, and disruption of the 4q35 D4Z4 array chromatin structure in somatic cells is associated with all forms of FSHD [25, 26]. These epigenetic disruptions can be environmentally influenced and are important therapeutic targets. Epigenetic disruptions in FSHD include chromatin relaxation through the SMCHD1 or DNMT3B gene defect causing FSHD type 2 (FSHD2) [13, 27], hypomethylation [14], alternative RNA splicing and nucleosome remodelling [15]. Investigating novel (epi)genetic characteristics in children with FSHD and linking this genetic profile to disease severity and age at onset will contribute to better predictors of prognosis and understanding of the pathogenesis.

### Clinical management

A highly needed evidence-based guideline for the diagnosis and management of FSHD has recently been published [28]. However, a specific guideline for children with FSHD and adult patients with early onset FSHD is currently lacking. Furthermore, adequate information on natural history and disease markers are important for clinical trial-preparedness [29], with fast approaching therapeutic trials [30–32].

In conclusion, data on the natural history, predictors and optimal clinical management of children with FSHD is limited. Thorough description of the clinical and genetic characteristics is vital for adequate management and could help to elucidate the underlying aetiology [31, 33]. Here we describe the objectives and methods of this population-based, prospective, observational study on early onset FSHD.

## Objectives

The primary objective of the study is:

1. to assess the clinical, genetic and epigenetic features of children with FSHD to optimize clinical management.
  The secondary objectives are:
2. to define a new comprehensive definition of early onset FSHD;
3. to provide prevalence estimations of early onset FSHD in The Netherlands;
4. to establish a well-characterized baseline cohort for prospective follow-up and recruitment for future clinical trials;
5. to assess 2-year disease progression rate in children with FSHD and develop a prognostic model.

## Methods and design

### Project design

The iFocus study is a prospective observational study performed at the department of paediatric Neurology of the Radboud University Medical Center, The Netherlands; it is a tertiary referral center for neuromuscular diseases. Genetic and epigenetic studies will be performed in the department of Human Genetics of the Leiden University Medical Center, The Netherlands. Participation in the study will not affect the usual care provided by the patients’ own medical team.

### Study population

Eligible patients are children aged 0–17 years with symptoms of facioscapulohumeral dystrophy and genetically proven FSHD1 or FSHD2 living in The Netherlands. Exclusion criteria are: inability or unwillingness to provide informed consent. The aim is to include all children in The Netherlands with a genetically confirmed diagnosis of FSHD. We expect to include 20–30 patients based on the estimated prevalence in the population [4].

There is no agreement on the classification of children with FSHD. Therefore we chose to include all patients less than 18 years with genetically confirmed FSHD, including for example children with isolated hearing loss as well. This enables us to describe the complete spectrum of children with FSHD. In addition, this study could identify predictors of severity and prognosis, thereby serving as a reference for working towards a widely accepted classification.

### Recruitment and screening

Participants will be recruited non-selectively and consecutively in the period from November 2015-November 2016. In order to estimate the prevalence of FSHD in children, we will try to reach all children with FSHD in The Netherlands through a very intensive recruitment. All Dutch paediatric neurologists, paediatricians and specialized paediatric physiotherapists will be personally asked about potential participants. In addition, all patients registered at the Dutch neuromuscular patient association [34] and the databases CRAMP [35] and Focus (NL48204.091.14) will be personally informed. Thirdly, we will directly recruit participants through promotion of our study on patient information days, patient websites and social media.

**Fig. 1:**
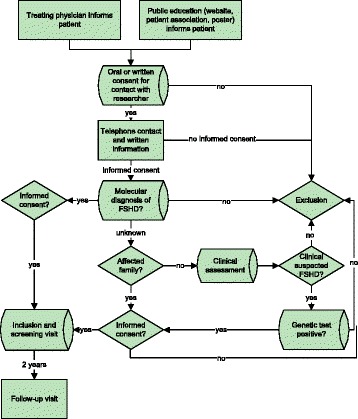
Flow chart of inclusion

In order to provide data on the complete spectrum of children with FSHD we aim to identify all children with clinically suspected FSHD, with or without genetic confirmation of FSHD. For this study we defined clinically suspected FSHD as: clinical weakness of the facial and/or upper-arm muscles for which the patient sought medical attention with exclusion of other diagnoses. For the children without genetic confirmation there are two scenarios:

1. Patients with clinically suspected FSHD and an FSHD-affected family member. Patients will be included in the study and genetic tests will be performed after obtaining informed consent.
2. Children with clinically suspected FSHD without an FSHD-affected family member will be invited for a pre-inclusion screening. This pre-screening will involve clinical examination by two of the authors (CE and RG). If FSHD is clinically highly suspected, the patient will be counselled for further assessments (i.e. genetic testing and referral to a specialized childhood neurologist for further rehabilitation care). If the diagnosis is genetically confirmed, the patient can be included in the study. If clinical suspicion is low or the genetic testing is negative, the children will not be included in the study and will be referred back to their treating medical staff (Fig. 1).

### Assessments

Participants will be invited for a baseline visit at the department of paediatric neurology at the Radboud University Medical Center. If the patient is unable to visit the hospital, the patient is offered a home visit and auditory, ophthalmologic and ultrasonography procedures will be omitted. Patients will have a follow-up visit after 2 years.

### Demographics

Date of birth, sex, weight (Kg), height (m) and medication will be recorded. Clinical characteristics of both parents will be directly examined and the genealogy of 3 consecutive generations will be drawn. The growth curves and medical history and medication use will be registered.

### Outcome measures

The outcome measures are listed in Table 1 and are structured according to the format of the International Classification of Functioning, Disability and Health for children and youth (ICF-CY [36]). The ICF is the World Health Organization framework for measuring health and disability at both individual and population levels and is subdivided in body functions, body structures, activities and participation and environmental factors. All procedures will be assessed at baseline visit and after 2-year follow-up if not otherwise specified.

**Table 1:** Outcome measures and tests at baseline and follow-up

Outcome domain	Tests	Age
Primary outcome measure		
ICF: Activities and participation		
Motor function	Motor Function Measure [37, 38]	2–18
Secondary outcome measures		
ICF: body functions		
Mental functions	Education level (school level)	5–18
	EEG [48]^a^	0–18
	Denver II developmental screening test [49]	0–6
Seeing functions	Visual acuity (Snellen)	4–18
Hearing functions	Tone- and speech audiometry	2–18
Pain	Faces scale pain [50]	6–18
Cardiac function	ECG [51]	0–18
Aerobic exercise tolerance	6-minute walk test MWT [52, 53]	walk
Fatigability	NeuroQol fatigue domain [54]	2–18
Respiratory function	Forced vital capacity and forced expiratory volume Spirometry [55]	6–18
Digestive, defecation, weight maintenance and urination functions	Qualitative history	0–18
Ingestion functions	TOMASS-C test [56]	6–18
	Neuromuscular disease swallowing status scale [57]	2–18
	Dysphagia questionnaire [40, 58]	2–18
Muscle functions	Handgrip dynamometer	6–18
	Manual muscle testing [59]	0–18
	Serum creatine kinase (CK)	0–18
	Age-adjusted clinical severity scale [60, 61]	5–18
	FSHD evaluation score [62]	
ICF: Body structure		
Eye structure	Slit lamp examination	4–18
	Dilated fundoscopy	4–18
	Optical coherence angiography tomography	6–18
Movement related structures	Muscle ultrasonography [39]	0–18
	Spine x-ray^a^	4–18
ICF: Activities and participation		
Communication/social interactions	Social Economic Questionnaire SEV [63]^a^	4–18
Self-care/life areas	Kidscreen [64]	8–18
ICF: Environmental factors		
Products and natural environment	Qualitative anamnesis and history	0–18

^a^ Only if clinically indicated

Outcome measures are listed in Table 1 and are extensive due to the explorative character of the study. The primary outcome is the motor function as measured by the Motor Function Measure [37, 38]. This measure specifically includes axial and upper limb functions, is designed for both ambulatory and non-ambulatory patients and can be continued into adulthood. Motor performance is supplementary assessed using the 6 min walking test, handgrip dynamometer and manual muscle testing MRC-scores. The FSHD evaluation score and the age-adjusted clinical severity scale are scored to explore the validity of these scales in children. In addition, non-muscular symptoms are extensively tested based on earlier literature on childhood onset FSHD [9–11, 17–19].

Quantitative muscle ultrasonography (QMUS) will be used to screen for muscle myopathy/inflammation. QMUS is a patient-friendly non-invasive method to screen for dystrophic changes in skeletal muscle [39]. The technique has been well-validated for screening and follow-up in several childhood and adult neuromuscular disorders [40]. The following muscles will be measured on both sides, using a fixed scanning protocol and settings as previously described [41, 42]: depressor anguli oris, masseter, sternocleidomastoideus, biceps brachii, rectus abdominis, rectus femoris, gastrocnemicus and tibialis anterior [43, 44].

Extramuscular symptoms will be assessed based on symptoms as reported in literature. This will include an extensive ophthalmologic screening, including optical coherence tomography with angiography for specific focus on the retinal arteries [45], a tone- and speech audiometry, electrocardiogram (ECG) and spirometry. Electro-encephalography (EEG) and spinal x-ray will be performed if indicated.

#### Prevalence

An improvement of the prevalence estimations will be assessed by comparing our recruitment with anonymous data of the genetic testing by the Leiden University Medical Center, where all Dutch samples are tested. This method is based on earlier research in the adult population [4].

#### (Epi)genetic disease-modifying factors

To explore the relationship between (epi)genetic disease-modifying factors and the disease severity, an extensive qualitative history is taken. Focus is on medical history (traumata, infections), dietary intake and maternal and perinatal health, including maternal substance use.

From participants and biological parents (if available) high molecular weight DNA and RNA will be isolated from peripheral blood mononucleated cell (PBMCs). The sizing of the D4Z4 repeats on chromosomes 4 and 10 will be executed using pulsed field gel electrophoresis (PFGE) and the haplotype analysis by hybridization of PFGE blots with probes A and B in combination with PCR-based SSLP analysis [21, 22]. Methylation of the D4Z4 repeat will be established at the FseI restriction site in the proximal unit of the D4Z4 array [26, 27]. In case of reduced CpG methylation at D4Z4, gene mutations in SMCHD1 or DNMT3B will be analysed by Sanger sequencing and predicted splice site mutations or nonsense mutations will be further analysed using cDNA isolated from PBMCs.

For RNA isolation from PBMCs we use PAXgene Blood RNA tubes which will be processed for obtaining high quality RNA for whole genome RNA sequencing analysis (RNAseq).

#### Cohort for future trial acquisition

An additional goal of this study is to obtain a well-documented cohort for longer (>2 years) follow-up and future trial acquisition. Patients will be asked to participate in the Dutch FSHD registration study [46] and for biomaterial storage. Serum samples of the participant and both biological parents will be stored in the Radboud biobank (http://www.radboudbiobank.nl/nl/) and are available for future DNA and/or RNA research for researchers worldwide.

### Data collection

All parents/legal guardians of participants will receive an online form with the standardized questionnaires. All participants > 8 years will also receive an online form with the standardized questionnaires for children. The forms will be sent through Castor and directly stored in the Castor database (Version 2015, [47]). All data-management and data-monitoring will be performed within the Castor software.

### Statistics

The Statistical Package for the Social Sciences (SPSS version 22, IBM, Armonk, New York) will be used to conduct all statistical analyses. Due to the explorative nature of the iFocus study, descriptive statistics will be applied to describe all patient characteristics. Continuous data are described as mean ± standard deviations. If reference values are available from literature, we will check for overlapping confidence intervals. Number of non-missing values is described (missing data are not replaced) unless stated otherwise. For describing the D4Z4 CpG methylation corrected repeat size, the delta 1 score will be used [14].

Correlations between (novel) molecular markers and clinical severity will be assessed with Spearman rank correlation. Possible correlations will be assessed on the following variables: ‘corrected repeat length’, ‘age at investigation’, ‘hereditary pattern’ ‘age at onset’, various extramuscular symptoms, FSHD severity score and motor function measurement scores. If novel (molecular) variables are found, these will be added in the statistical model. To minimize the multicolinearity manual backward reduction will be executed.

For the clinical prediction model, multiple linear regressions will be used to explore the relationship between potential disease modifying variables and disease severity. In addition, for the 2 year follow-up data, linear mixed models will be applied for analysis of differences in disease progression. The primary outcome is a continuous variable, FSHD clinical severity score. The fixed effect predictors are repeat length (divided in 1–3 D4Z4 repeat units (U), 4–6 U and 7–10 U), current age, hereditary pattern and sex.

### Time plan

Patients will be included in 2016 and follow-up visits will be scheduled in 2018. The first results are expected at the end of 2018.

## Discussion

Results from the iFocus study will provide insights into the clinical and genetic spectrum of children with FSHD. These insights are vital for adequate symptomatic management and clinical trial-readiness. We propose extensive qualitative and quantitative measurements specified by age and use a modified format of the International Classification of Functioning, Disability and Health criteria. This structured approach enables us to give an explorative, full-spectrum, unbiased clinical description of FSHD in children.

Furthermore, this study aims to provide additional insight in the epigenetic and environmental disease modifying factors. This could help to unravel the aetiology of FSHD and serve as a basis for prognostic and therapeutic models.

One of the study’s strengths is the excellent recruitment organisation in The Netherlands, thereby reducing selection bias. There is a well-organized health system with free access to paediatric neurology for every child. In addition, our centers have a longstanding history of excellent FSHD research and administration. Finally, all participants can reach the study center within three hours. These assets make it possible to execute a population based, nation-wide study.

A critical issue is the small number of participants, making it hard to verify correlations or differences in subgroups, international collaborations are in preparation. Although, we expect to find differences in motor functioning after 2 years of follow-up, this period will possibly be too short for changes in other outcome measures such as wheelchair dependency. Therefore we aim to continue the follow-up of this cohort for a longer period of time.

In conclusion, the iFocus study is expected to yield new knowledge on children with FSHD, aiming to optimize clinical management, help unravelling the aetiology of FSHD and serve as a basis for prognostic and therapeutic studies.

## Abbreviations

DNA: Deoxyribonucleic acid
DNMT3B: DNA methyltransferase 3B
FSHD: Facioscapulohumeral muscular dystrophy
ICF-Y: The International Classification of Functioning, Disability and Health for children and youth
MFM: Motor Function Measure
MRC: Medical Research Council
PBMC: Peripheral blood mononucleated cell
PFGE: Pulsed field gel electrophoresis
RNA: Ribonucleic acid
SMCHD1: Structural maintenance of chromosomes flexible hinge domain containing 1
U: D4Z4 repeat unit
WHO: World Health Organisation
